# Investigations on incidence and relevant factors of allergies in 5725 urban pregnant women: a cohort study in China

**DOI:** 10.1186/s12889-022-14355-7

**Published:** 2023-01-18

**Authors:** Gao Qun, Sun He, Song Bo, Di Jiangli, Xu Tao, Wang Shuo, Lu Zechun, Wang Ailing

**Affiliations:** National Center for Women and Children’s Health Chinese Center for Disease Control and Prevention, Beijing, China

**Keywords:** Allergies, Pregnant women, Cohort study, Relevant factors

## Abstract

**Background:**

Allergic diseases are highly prevalent in the women of childbearing age. As we know, the immune system could change when pregnancy, which may affect the course of allergic diseases. Meanwhile, they also can affect the course and outcome of pregnancy. The data on incidence of allergies during pregnancy is lacking and conducting clinical trials in pregnant women was limited, therefore, we observed a prebirth cohort to supplement the relevant data and strengthen concerned research conductions.

**Objective:**

We aim to obtain the incidence of allergies in urban pregnancy and explore the relevant factors of allergic diseases in urban pregnancy.

**Methods:**

We design a multicenter and prospective cohort in 20 institutions above municipal level which were eligible according to the study design from 14 provinces covering all-side of China. This cohort was conducted from 13^+6^ weeks of gestation to 12 months postpartum and in our study, we chose the prenatal part to analyze. The outcome was developing allergies during pregnancy, which were diagnosed by clinicians according to the uniform criterion from National Health Commission. All the data was collected by electronic questionnaires through tablet computers.

**Results:**

The incidence of allergic diseases in urban pregnant women was 21.0% (95%*CI* 20.0% ~ 22.0%). From social demography data, the history of allergies of pregnant women and their parents had statistical significance(*p* < 0.01); For exposure to living or working environment, house decoration for less than half a year, exposure to plush toys, disinfectants, insecticides, antihistamines, glucocorticoids, antipyretic analgesics, tocolytic agent and probiotics had statistical significance (all *p* < 0.05); For psychological status, self-rated depression and anxiety had statistical significance (*p* = 0.026;*p* = 0.006).

**Conclusion:**

The incidence of allergic diseases in urban pregnant women was similar to the former study and kept a medium–high level. The history of allergies of pregnant women and their parents, house decoration time, exposure to plush toys, disinfectants, insecticides, antihistamines, glucocorticoids, antipyretic analgesics, tocolytic agents, probiotics, self-rated depression, and anxiety were relevant factors of allergic diseases during pregnancy.

## Introduction

As one of the non-communicable diseases, allergy is dramatically emerging in both developing and developed countries. Allergy is a hypersensitivity reaction initiated by specific immunologic responses against foreign, usually harmless, substances. The incidence of allergic diseases is on the rise all over the world and allergies have become a serious public health problem and caused a severe disease burden [1]. Nowadays, around 20% of pregnant women are effected with allergic diseases, especially rhinitis and asthma [2]. Others like atopic dermatitis, allergic conjunctivitis, acute urticaria, food allergy, and drug allergy can also occur frequently in pregnant women. These conditions make pregnancy more complex, for example, women with asthma when pregnant have higher risks of prematurity, small for gestational age (SGA), and neonatal intensive care unit admission [3]. Allergy not only causes long-term immune dysfunction but also has underlying inflammation, which forms the underlying factor for other non-communicable diseases [4]. Recent studies found that maternal nutrition, pollutant particles and smoking or exposure to smoke can influence the incidence of allergy in offspring [5–7].

Normally, the risk factors associated with allergic diseases include genetic factors and environmental factors [8]. Previous researches mostly focus on the influence factors of allergic diseases in adults but not pregnancy. To date, there is no comprehensive evidence quantifying the plausibility of the association between developing allergic diseases and influence factors in pregnant women. We conducted a large cohort lasting from November 2017 to July 2020 with the outcomes of infants’ food allergies in 20 institutions above municipal level covering 14 provinces in China. And in this article, we explore the detailed relevant factors of allergy in pregnancy, which is part of the large cohort. Particularly, in addition to sociodemographic characteristics, intake of food and possible exposure, we also collect information about psychological status like depression and anxiety.

## Material and Method

### Study objectives

- 1.1.1 To master the incidence of various allergies among pregnant women in part urban areas of China;
- 1.1.2 To know the relevant factors that associated with allergies during pregnancy;

### Study sites and participants

According to the geographical and population distribution of China, 20 municipal and provincial maternal and child health hospitals(MCH) from 14 provinces (covering the east, west, north and south) were selected as research institutions, which had the conditions for the diagnosis of allergic diseases, or the ability to be transferred to the local general medical institutions with allergic diagnosis function. Simultaneously, all the MCH institutions could follow up with the study participants standardly in succession. As for the large cohort, sample size was calculated according to the rate of the infant food allergies (8%) cohort. Meanwhile, we used 5–20 times the number of independent variables to be studied and the incidence of allergic rhinitis (30%), asthma (23.2%-32%) and urticaria (20%) in population mentioned in the literature [9–11] to verify the sample size. The completed sample size(5725) was representative and met the requirements. Following written informed consent, pregnant women completed the first questionnaire at enrollment in the first trimester of pregnancy (13^+6^ weeks). They should have been living in the city where the research institution is located for more than one year and plan to continue to live there for more than two years, therefore, investigators can follow up with them in the third trimester.

### Study procedure

**Fig. 1:**
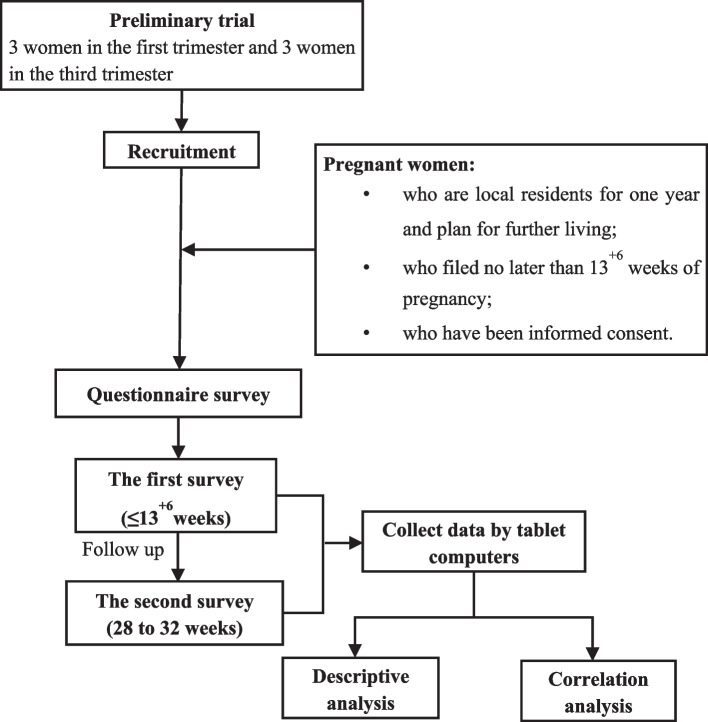
Trail design

After conducting the preliminary trial which selected 3 women in the first trimester and 3 in the third trimester, questionnaires were modified by experts and professors and then administrated to all participating women by trained investigators in both the first trimester (under 13^+6^ weeks) and third trimester (between 28 to 32 weeks) face to face. Relevant information contained demographic characteristics, history of allergies, residential and working environment, lifestyle, gestational age, complications of pregnancy, medication and psychological status during pregnancy. The outcome variable is whether the participants developed allergies during pregnancy which were diagnosed by clinicians according to uniform criterion [12–18]. The first and follow-up investigations were conducted in person at participating hospitals or maternal and child health care institutions covering 14 provinces in China. The detailed procedure was presented in Fig. 1.

### Data collecting

Data collection is mainly completed through tablet computers and summarized in a specialized electronic database managed by National Center for women and children’s health. With the unified information system, electronic questionnaire and related data entry, storage, logical error correction, data generation, submission and upload procedures were more efficient. All the data was collected by two people who had been trained before.

### Statistical analyses

To master the incidence of allergic diseases in pregnant women, we counted occurrence times from the first to third trimesters. Descriptive statistics were used to study the demographic and general information of the participants. *χ*^*2*^ test and exact Fisher test were used to analyze various factors whether had difference between the allergic group and the control group [19]. The tests were two-tailed with the threshold of significance at *P* < 0.05, *P* values less than 0.05 were considered significant. We performed all analyses using STATA 14.0.

## Results

A total of 5725 pregnant women were recruited in the study from 20 institutions from 14 provinces of China.

### Baseline information

#### Demographic characteristics of pregnant women

Table 1 shows general information of participants and their family, including age, ethnic group, pre-pregnancy body mass index (Pre-BMI), occupation, highest education level, per capita household income, etc.

**Table 1:** Baseline information

	**Numbers (n)**	**Constituent ratio (%)**		**Numbers (n)**	**Constituent ratio (%)**
**2.1.1 Demographic characteristics of pregnant women**					
**Age**			**Ethnic group**		
< 35	4898	(85.6)	Han	5423	(94.7)
≥ 35	827	(14.4)	Minority	302	(5.3)
**Pre-BMI**					
< 18.5(Underweight)	928	(16.2)			
18.5 ~ 23.9(Normal weight)	3946	(68.9)	**highest education level**		
24 ~ 28(Overweight)	647	(11.3)	Postgraduate and above	470	(8.2)
> 28(Obesity)	204	(3.6)	College/university degree	4059	(70.9)
**Per capita monthly household income (RMB)**			High school/equivalent high school diploma and below	1196	(20.9)
< 1000	22	(0.4)			
1000 ~ < 3000	218	(3.8)			
3000 ~ < 5000	1295	(22.6)			
5000 ~ < 10,000	2323	(40.6)			
10,000 ~ < 30,000	1629	(28.4)			
≥ 30,000	238	(4.2)			
**2.1.2 History of allergies of pregnant women and family members**					
**Pregnant women**					
**Pre-existing allergies**			**Classification of allergy history**		
Yes	1383	(24.2)	One	1304	(22.8)
No	4342	(75.8)	Two	288	(5.0)
			More than three	87	(1.5)
			None	4045	(70.7)
**Fathers**			**Mothers**		
**Pre-existing allergies**			**Pre-existing allergies**		
Yes	343	(6.0)	Yes	390	(6.8)
No	5382	(94.0)	No	5335	(93.2)
**Classification of allergy history**			**Classification of allergy history**		
One	319	(93.0)	One	352	(90.3)
Two	22	(6.4)	Two	29	(7.4)
More than three	2	(0.6)	More than three	9	(2.3)
**2.1.3 Exposure in living or working environment of pregnant women**					
**Exposure to allergens when working**			**Planting**		
Yes	167	(2.9)	Yes	3288	(57.4)
No	5558	(97.1)	No	2437	(42.6)
**Decorations**			**Pets contacting**		
< Half a year	95	(1.7)	Never	4852	(84.7)
Half a year ~ < Two years	1191	(20.8)	Occasional contact (1–2	521	(9.1)
≥ Two years	4439	(77.5)	times/month)		
			Frequent contact (not close)	255	(4.5)
**Carpet**			Close contact (daily contact)	97	(1.7)
Yes	575	(10.0)			
No	5150	(90.0)	**Plush toys**		
**Main cooking fuel**			Yes	1104	(19.3)
Fuel gas	2061	(36.0)	No	4621	(80.7)
Electricity	3538	(61.8)	**Disinfectants**		
Coal	126	(2.2)	Yes	601	(10.5)
**Ventilation system**			No	5124	(89.5)
Natural ventilation	545	(9.5)	**Mosquito repellents**		
Fans	299	(5.2)	Yes	330	(5.8)
Air-conditions	4881	(85.3)	No	5395	(94.2)
**Heating facilities**			**Insecticides**		
Heating radiator	806	(14.1)	Yes	45	(0.8)
Air-conditioner	3556	(62.1)	No	5680	(99.2)
Stove	1363	(23.8)	**Air fresheners**		
**Home humidity**			Yes	175	(3.1)
Moist	70	(1.2)	No	5550	(96.9)
Moderate	4634	(81.0)			
Dry	1021	(17.8)			
**2.1.4. Smoking and alcohol use among pregnant women**					
**Smoking**			**Drinking frequency**		
Active smoking	28	(3.0)	Everyday	2	(0.7)
Passive smoking	908	(97.0)	Every week	12	(4.3)
Total	936	(100.0)	Every month	268	(95.0)
			Total	282	(100.0)
**2.1.5 Medication and probiotics intake among pregnant women**					
**Medication intake**			**Progesterone-type tocolytic**		
Yes	2525	(44.1)	**agents**		
No	3200	(55.9)	Yes	2320	(2.7)
**Antihistamines**			No	3405	(97.3)
Yes	44	(0.8)	**Single or combined medication**		
No	5681	(99.2)	Only one	2268	(89.8)
**Antibiotics for 3 consecutive days**			Two at once	216	(8.6)
Yes	233	(4.1)	Three or more at once	41	(1.6)
No	5492	(95.9)	**Probiotics**		
**Glucocorticoids**			Everyday	40	(5.2)
Yes	76	(1.3)	≥ 3 days every week	261	(34.1)
No	5649	(98.7)	≤ 2 days every week	98	(12.8)
**Antipyretic analgesics**			1 ~ 3 days every month	366	(47.9)
Yes	153	(2.5)	Total	765	(100.0)
No	5572	(97.5)			

### History of allergies of pregnant women and family members

We also list the history of allergies in pregnant women and their parents in Table 1, mainly about (1) asthma; (2) allergic rhinitis; (3) atopic dermatitis (itchy inflammation of the skin, with a tendency to flare up from time to time); (4) eczema; (5) food allergy; (6) drug allergy; (7) allergic conjunctivitis; (8) Allergic urticaria (9) others, for example, contact dermatitis, ultraviolet allergy (solar dermatitis), allergic cheilitis, allergic purpura, positive skin scratch sign, allergic bronchitis, etc. All these allergic diseases mentioned above were diagnosed by clinician from different departments.

### Exposure in residential or working environment of pregnant women

Pregnant women who may expose to the high-risk allergens when working account for 2.9% (167). The allergens generally contained rubber, paints/coatings, adhesives, chemical plastics, pesticides, leather/fur products, while in living conditions, the decoration, carpet, cooking fuel use, heating, ventilation, home humidity, plants breeding (pollen contact), pets, plush toys, disinfectants, mosquito repellents, insecticides, air fresheners and other substances all had the possibility to cause allergies. The detailed data can be found in Table 1.

### Smoking and alcohol use among pregnant women

According to investigations, a total of 936 (16.3%) of 5725 pregnant women were exposed to smoking in their daily life and work environment, which were divided into active smoking and passive smoking. The passive smoking can be found because their husbands, their colleagues, their parents or their other family members were smokers. As for drinking during pregnancy, only 4.9% admitted they had drunk, that’s to say 282 pregnant women.

### Medication and probiotics intake among pregnant women

We surveyed the using frequency of several medication that have been shown in the literature to have effect on allergies, including antihistamines, antibiotics, glucocorticoids, antipyretic analgesics and progesterone-type tocolytic agent. The results demonstrated that there were 2525 cases (44.1%) of pregnant women who had taken these drugs and 765 (13.4%) who had received probiotics.

### Mental state of pregnant women

We use Patient health questionnaire 9(PHQ-9) scale for depression and Generalized anxiety disorder-7(GAD-7) scale for anxiety, each scale was assigned a score according to which the diagnosis and grading of disease were assisted. The occurrence of depression/anxiety in either first or last trimester or both stages was considered as the occurrence of depression/anxiety, and the higher score was regarded as the grading standard when both stages occurred. The score and grading of depression scale and anxiety scale are shown in Table 2.

**Table 2:** The score and grading of PHQ-9 and GAD-7

PHQ-9 for depression	
< 5 points	Normal
5 ~ 9 points	Mild
10 ~ 14 points	Moderate
15 ~ 19 points	Less severe
≥ 20 points	Severe
**GAD-7 for anxiety**	
0 ~ 4 points	Normal
5 ~ 9 points	Mild
10 ~ 14 points	Moderate
15 ~ 21 points	Severe

According to the PHQ-9, 3955 pregnant women (69.1%) with different degrees of depression were found, mild depression was the highest (76.2%); The results of the GAD-7 showed that 2032 pregnant women (35.5%) had different degrees of anxiety during pregnancy, and mild anxiety accounted for the highest proportion (85.8%). More details were found in Table 3.

**Table 3:** Grading of depression and anxiety among pregnant women

	**Numbers (n)**	**Constituent ratio (%)**
**Depression**		
Mild	3013	76.2
Moderate	736	18.6
Less severe	165	4.2
Severe	41	1.0
Total	3955	100.0
**Anxiety**		
Mild	1744	85.8
Moderate	244	12.0
Severe	45	2.2
Total	2032	100.0

### Incidence of allergic diseases in pregnant women

**Fig. 2:**
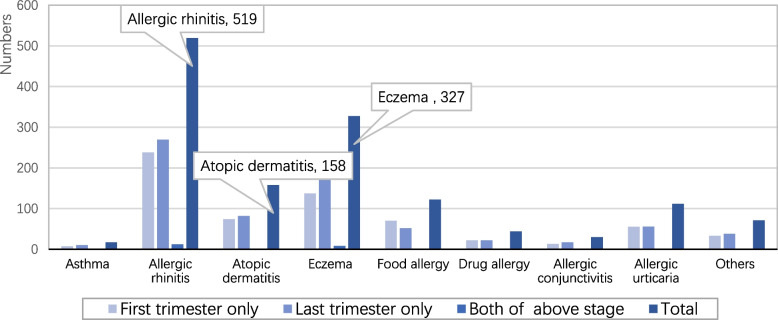
Quantities of different allergies at different stages of pregnancy

Among 5725 participants who have been followed up completely, 1200 pregnant women developed allergies that were diagnosed by clinician in anytime of pregnancy. The incidence was 21%(95%Confidence Intervals[*CI*]:20.0% ~ 22.0%). As shown in Table 4, the number, incidence and proportion of allergies in pregnant women at different stages of pregnancy were various. We found the proportion of pregnant women with allergy only in the third trimester was the highest (53.3%). In addition, the incidence of different categories of allergies in pregnancy was not unified. We can see the highest three were 519 cases of allergic rhinitis (9.07%), 327 cases of eczema (5.71%) and 158 cases of atopic dermatitis (2.76%), more details are listed in Fig. 2. Table 5 depicts single or combined occurrence of allergies during pregnancy, results indicate that single occurrence was most common, accounting for 18.6% of all allergic cases.

**Table 4:** Incidence of allergies at different stage of pregnancy

Stage	Numbers(n)	Incidence(%)	Constituent ratio
First trimester only	538	9.4	44.8
Last trimester only	639	11.2	53.3
Both of above stages	23	0.4	1.9
Total	1200	21.0	100.0

**Table 5:** Single or combined occurrence of allergies during pregnancy

Number of allergies	Numbers (n)	Incidence (%)	Constituent ratio (%)
Single occurrence	1065	18.6	88.8
Two kinds simultaneously	119	2.1	9.9
Three kinds simultaneously	16	0.3	1.3
Total	1200	21.0	100.0

### Correlation analysis between risk factors and allergies in pregnant women

We divided two groups by whether participants had allergies during pregnancy, which were allergic group (1200 cases) and control group (4525 cases). By univariate analysis of *χ*^*2*^ test and Fisher’s Exact Test, we analyzed differences of factors between two groups and explored whether the affect from above factors may change the incidence of allergies during pregnancy. The results showed that relevant factors contains that the history of allergies of pregnant women and their parents, house decoration time, exposure to plush toys, disinfectants, insecticides, antihistamines, glucocorticoids, antipyretic analgesics, tocolytic agent, probiotics, self-rated depression and anxiety. There were statistically significant differences between the two groups (*P* < 0.05) when analyzing those influence factors (Table 6).

**Table 6:** Univariate analysis of allergy in pregnant women

	**Allergic group**		**Control group**		***χ***^***2***^	***P* value**
	**Number**	**Ratio (%)**	**Number**	**Ratio (%)**		
**Age**						
< 35	1026	85.5	3872	85.6	0.004	0.952
≥ 35	174	14.5	653	14.4		
**Ethnic group**						
Han	1150	95.8	4273	94.4	3.733	0.053
Minority	50	4.2	252	5.6		
**Pre-existing allergies of pregnant women**						
Yes	563	46.9	820	18.1	429.241	0.000
No	637	53.1	3705	81.9		
**Pre-existing allergies of their fathers**						
Yes	141	11.8	229	5.1	70.167	0.000
No	1059	88.2	4296	94.9		
**Pre-existing allergies of their mothers**						
Yes	138	11.5	252	5.6	52.556	0.000
No	1062	88.5	4273	94.4		
**Carpet**						
Yes	121	10.1	454	10.0	0.003	0.959
No	1079	89.9	4071	90.0		
**Heating facilities**						
Heating radiator	176	14.7	630	13.9	2.151	0.341
Air-conditioner	757	63.1	2799	61.9		
Stove	267	22.2	1096	24.2		
**Ventilation**						
Yes	106	8.8	436	9.6	0.712	0.399
No	1094	91.2	4089	90.4		
**Cooking fuel use**						
Yes	790	65.8	2874	63.5	2.215	0.137
No	410	34.2	1651	36.5		
**Home humidity**						
Moist	12	1.0	58	1.3	1.030	0.597
Moderate	966	80.5	3668	81.1		
Dry	222	18.5	799	17.6		
**Decorations**						
< Half a year	27	2.3	68	1.5	6.042	0.049
Half a year ~ < Two years	269	22.4	922	20.4		
≥ Two years	904	75.3	3535	78.1		
**Pets contacting**						
Never	993	82.8	3859	85.3	5.064	0.167
Occasional contact (1–2 times / month)	120	10.0	401	8.9		
Frequent contact (not close)	63	5.3	192	4.2		
Close contact (daily contact)	24	2.0	73	1.6		
**Planting**						
Yes	712	59.3	2576	56.9	2.244	0.134
No	488	40.7	1949	43.1		
**Plush toys**						
Yes	262	21.8	842	18.6	6.340	0.012
No	938	78.2	3683	81.4		
**Disinfectants**						
Yes	167	13.9	434	9.6	18.887	0.000
No	1033	86.1	4091	90.4		
**Mosquito repellents**						
Yes	70	5.8	260	5.7	0.013	0.908
No	1130	94.2	4265	94.3		
**Insecticides**						
Yes	15	1.25	30	0.7	4.191	0.041
No	1185	98.75	4495	99.3		
**Air fresheners**						
Yes	37	3.1	138	3.0	0.004	0.952
No	1163	96.9	4387	97.0		
**Smoking**						
Yes	993	82.75	3796	83.9	0.901	0.343
No	207	17.25	729	16.1		
**Drinking**						
Yes	62	5.2	220	4.9	0.187	0.655
No	1138	94.8	4304	95.1		
**Antihistamines**						
Yes	11	0.9	19	0.4	4.490	0.034
No	1189	99.1	4506	99.6		
**Antibiotics**						
Yes	33	2.75	86	1.9	3.362	0.067
No	1167	97.25	4439	98.1		
**Glucocorticoids**						
Yes	18	1.5	22	0.5	14.051	0.000
No	1182	98.5	4503	99.5		
**Antipyretic analgesics**						
Yes	32	2.7	62	1.4	9.872	0.002
No	1168	97.3	4463	98.6		
**Tocolytic agent**						
Yes	422	35.2	1414	31.2	6.684	0.010
No	778	64.8	3111	68.8		
**Probiotics**						
Yes	89	7.4	236	5.2	8.583	0.003
No	1111	92.6	4289	94.8		
**Depression grading**						
Normal	332	27.7	1438	31.8	11.040	0.026
Mild	673	56.1	2340	51.7		
Moderate	156	13.0	580	12.8		
Less severe	28	2.3	137	3.0		
Severe	11	0.9	30	0.7		
**Anxiety grading**						
Normal	712	59.3	2932	64.8	12.392	0.006
Mild	416	34.7	1357	30.0		
Moderate	62	5.2	199	4.4		
Severe	10	0.8	36	0.8		
**Pre-BMI**						
18.5 ~ 23.9(Normal weight)	846	70.5	3100	68.5	1.939	0.379
< 18.5(Underweight)	181	15.1	747	16.5		
24 ~ 28(Overweight)	127	10.6	520	11.5		
> 28(Obesity)	46	3.8	158	3.5		

## Discussion

Nowadays, the health quality of pregnant women and their offspring were attached high importance as it related to health development of the two generations. This study kept attention to allergic diseases during pregnancy, the relevant data of which was nearly scarce in China and even Asia. According to the results, we found 1200 participants (21%) developed allergies during pregnancy in urban area, which was similar to the result of Isabella et al*. *[2] from secondary publication. The highest three kinds of allergies were allergic rhinitis(9.07%), eczema(5.71%) and atopic dermatitis(2.76%). The incidence of allergic rhinitis was lower than in recent surveys, Orban et al*. *[20]conducted and concluded the pregnant rhinitis was 18% ~ 30%, while in Turkish [21]women was 17.17%. Gestational itchy dermatoses which contained eczema and atopic dermatitis are relatively common, with eczema being diagnosed in 36% to 49% of all pregnancy dermatoses [22]. Our findings showed the incidence of asthma during pregnancy was 0.3%, which was quite different from a worldwide survey that explained asthma affects up to 13% of pregnancies worldwide [23] and asthma prevalence is increasing [24, 25]. The possible reason for the discrepancy between our findings and other study was that diagnostic criteria and survey methods had differences. In the light of our study design, we utilized special electronic questionnaires and a unified electronic database which could ensure the integrity and authenticity of data, simultaneously, the large sample size covering 14 provinces in China could almost represent the race/ethnicity, living habits and environment of the Chinese urban females.

Our study could not only supplement data on allergies of urban pregnant women but increase the knowledge and understanding of the relevant factors of allergic diseases during pregnancy. Our findings on the correlation between various factors and allergies during pregnancy are in accordance with the previous studies, for example, the history of allergies was confirmed in a cohort study in a hospital in the USA [26]. Environment elements particularly indoor substances (decorations, disinfectants, insecticides, *et. al*), medication (glucocorticoids, antipyretic analgesics, tocolytic agent, *et. al*), and psychological stress were mentioned [10, 27, 28] and demonstrated association with allergies. These findings will enable physicians to better counsel families with allergic diseases and enable researchers to develop targeted prevention strategies. We also found drinking had influence on allergies but exposure to smoke (passive or active) had no significance, which contradicted the results from Pali-Schöll et al*. *[8] that exposure to smoke was a risk factor of allergy. Even so, excessive drinking or smoking was not recommended during pregnancy, which may lead to a variety of chronic diseases but not limited to allergies. Recently, probiotics supplementation is a popular theme to prevent allergies [29], and current available evidence indicates the net benefit of eczema prevention. Likewise, we found probiotics supplementation can mildly influence the risk of allergy. This suggested further research should be conducted to find the detailed causality of probiotics and allergies and maybe indicate the new prevention method of controlling allergic diseases during pregnancy.

It is worth noting that we found depression and anxiety both had an association with allergies during pregnancy (*p* = 0.026, *p* = 0.006 respectively). In accordance with this result, the study in the US [30] and Japan [31]found that food allergy and atopic dermatitis were more common in depressed people. Li et al*. *[32] reported that anxiety is associated with greater perceived dyspnea in asthma. The literature demonstrated that pregnant women affected by psychological disorders such as anxiety and depression are universal(15.04% and 5.19% [33]), which made a high risk of allergic diseases. Thus doctors and family members should terribly concern about the mental health of pregnant women in order to reduce the threat of allergic diseases.

The data of our study came from following participants in a longitudinal prebirth cohort, obtaining information prospectively. Therefore, we obtained the causal relationship between multiple factors and allergies outcome in a rational chronological order. While some limitations cannot be neglected. To begin with, as a large cohort, loss to follow-up is irresistible for the reasons like spontaneous abortion, withdrawal and move during study, while the sample was representative according to the validation we made before analysis. We would also consider including spontaneous abortion in the first trimester in the subsequent study to explore the influence of them. Secondly, we conducted the cohort for approximately two years and some factors may change during follow-up, which made information biased in the analysis. Further survey about risk or protective factors of allergic diseases and multivariate analysis should continue being carried out. In addition, we were unable to define the different clinical allergic diseases into subtypes despite the outcomes of the participants were confirmed by clinicians.

## Conclusion

Our study demonstrated the incidence of allergic diseases in urban pregnant women was 21.0% (95%*CI*: 20.0% ~ 22.0%), which kept a medium high level, and the relevant factors of allergies during pregnancy contained the history of allergies of pregnant women and their parents, house decoration for less than half a year, exposure to plush toys, disinfectants, insecticides, antihistamines, glucocorticoids, antipyretic analgesics, tocolytic agent, probiotics, self-rated depression and anxiety. Further study had been taken into consideration. Firstly a survey in rural areas would be carried out to investigate the occurrence of allergies in rural pregnant women in China. Next, analysis of risk factors of allergies during pregnancy should be taken to explore the mechanism of allergy in pregnant women. And intervention studies should be conducted to reduce the incidence of maternal allergies.

## Data Availability

The datasets used and/or analyzed during the current study are available from the corresponding author on reasonable request.

## Abbreviations

SGA: Small for gestational age
MCH: Maternal and child health hospitals
Pre-BMI: Pre-pregnancy body mass index
PHQ-9: Patient health questionnaire 9
GAD-7: Generalized anxiety disorder-7
CI: Confidence Intervals
